# Density of Deep Eutectic Solvents: The Path Forward Cheminformatics-Driven Reliable Predictions for Mixtures

**DOI:** 10.3390/molecules26195779

**Published:** 2021-09-24

**Authors:** Amit Kumar Halder, Reza Haghbakhsh, Iuliia V. Voroshylova, Ana Rita C. Duarte, M. Natalia D. S. Cordeiro

**Affiliations:** 1 LAQV@REQUIMTE/Department of Chemistry and Biochemistry, Faculty of Sciences, University of Porto, 4169-007 Porto, Portugal; amit.halder@fc.up.pt (A.K.H.); voroshylova.iuliia@fc.up.pt (I.V.V.); 2 LAQV@REQUIMTE/Department of Chemistry, Faculty of Sciences and Technology, New University of Lisbon, 2829-516 Caparica, Portugal; haghbakhsh@gmail.com (R.H.); ard08968@fct.unl.pt (A.R.C.D.)

**Keywords:** DES, density, cheminformatics, QSPR, validation, consensus modelling, thermophysical properties

## Abstract

Deep eutectic solvents (DES) are often regarded as greener sustainable alternative solvents and are currently employed in many industrial applications on a large scale. Bearing in mind the industrial importance of DES—and because the vast majority of DES has yet to be synthesized—the development of cheminformatic models and tools efficiently profiling their density becomes essential. In this work, after rigorous validation, quantitative structure-property relationship (QSPR) models were proposed for use in estimating the density of a wide variety of DES. These models were based on a modelling dataset previously employed for constructing thermodynamic models for the same endpoint. The best QSPR models were robust and sound, performing well on an external validation set (set up with recently reported experimental density data of DES). Furthermore, the results revealed structural features that could play crucial roles in ruling DES density. Then, intelligent consensus prediction was employed to develop a consensus model with improved predictive accuracy. All models were derived using publicly available tools to facilitate easy reproducibility of the proposed methodology. Future work may involve setting up reliable, interpretable cheminformatic models for other thermodynamic properties of DES and guiding the design of these solvents for applications.

## 1. Introduction

Over the last few decades, demand has sharply increased for the replacement of toxic organic chemicals with more environmentally safe alternatives [1,2], This led to the emergence of green solvents, such as ionic liquids (ILs) and deep eutectic solvents (DES) [3,4,5,6]. However, as far as ecotoxicity is concerned, DES have been found to be more eco-friendly than ILs [7,8,9]. In fact, they are not only greener than ILs, they are less expensive. The price, eco-friendliness, non-volatile nature, biodegradability, and ease of preparation all make DES one of the most desirable and well-investigated industrial solvents [10,11]. A suitable combination of a hydrogen bond acceptor (HBA) with a hydrogen bond donor (HBD) in a specific molar ratio gave rise to a DES with a freezing point considerably lower than each of its components [5,6,12]. There have been reports of mixing two HBDs at the same time to achieve formation of the so-called ternary DES, but the latter was deemed out of the scope of the present study [13].

Similar to other industrial solvents, the density of DES is a commonly investigated physicochemical property, frequently needed in process design and optimization [1,2]. Likewise, knowledge of the ways temperature and pressure influence DES density is often required for finding suitable equations of states, which in turn help in establishing their industrial applications [6,14,15]. The density of DES can vary substantially depending on the nature and concentration of their constituents; however, most DES are denser than water [6]. To date, only a few thermodynamic models have been reported on the density of DES. Recently, we reported a simple and global thermodynamic model—based on critical temperature, critical volume, acentric factor, and measuring temperature—for estimating the density of a wide range of DES [14]. Herein, our aim was to explore cheminformatic modelling techniques to derive predictive models for characterizing the density of diverse DES.

Quantitative structure property relationship (QSPR) is a long-utilized cheminformatic techniques that has often been applied to predict the physicochemical properties of a large range of chemicals [16,17,18]. Despite the significant number of QSPR modelling studies targeting predictions of the density of ILs (predecessors of DES) [19], to the best of our knowledge, only two QSPR studies, both based on the COSMO-RS approach, have been reported so far (by Lemaoui et. al.) for predicting the density of DES [20,21]. The first study [20] was based on hydrophilic DES, whereas the second, more recent one [21] focused solely on hydrophobic DES. However, both studies pertained to a smaller number of data points compared to those handled herein. Furthermore, both lacked an in-depth validation of the developed models—which is considered crucial for QSPR modelling of mixtures (see Section 2.3.)—which restricted their overall applicability. The main aim of the present work was to set up linear, interpretable, highly predictive, and properly validated QSPR models for characterizing the density of a wide range of hydrophilic DES, following the principles of the Organization for Economic Cooperation and Development (OECD). According to the OECD, the following five requirements must be met in order for a QSPR study to be accepted: (i) a well-defined end point; (ii) an unambiguous algorithm; (iii) a defined applicability domain; (iv) suitable measures of goodness of fit, robustness and validation; and (v) a mechanistic interpretation, if possible [22]. Yet the scope of this work was not solely limited to such an aim; it also dealt with solving challenges related to cheminformatic analysis of mixtures in a simple and straightforward fashion, using in-house, open-access tools. Thus, the methodology applied here may be extended in the future to other thermodynamic properties of DES.

## 2. Materials and Methods

### 2.1. Dataset Collection

Undoubtedly, selection of dataset is not only the first, but also the most important step in cheminformatic analyses. In the present work, we selected a dataset containing 145 DES with 1154 data points collected from our previous work [14], wherein the development of a thermodynamic model for DES density was reported. This dataset assembled the experimental densities (in g/cm^3^) of a wide range of DES, measured in the temperature range from 283.15 K to 373.15 K at ambient pressure. In addition to being reliable for finding structural requirements for DES density estimation, these data allow for consideration of temperature as an independent parameter and evaluation of its relation to density. The large variation of chemicals (i.e., 17 types of HBAs and 42 types of HBDs) also made this dataset suitable for developing predictive and reliable QSPR models. Nevertheless, the dataset was updated by including all recent data reported in literature after publication of our previous work, i.e., since 2019. For this purpose, new, experimentally determined density values—measured under the same temperature and pressure conditions—were collected from recently published literature. This new dataset contained a total of 207 new data points, including five HBAs and three HBDs not present in the initial modelling dataset. However, instead of merging this new data with the old, we decided to maintain the old dataset (*n* = 1154) as the modelling dataset and the new dataset (*n* = 207) as an additional validation set, henceforth referred as the external validation set. Thus, the modelling dataset was used for identifying and establishing the most predictive QSPR models, whereas the external validation set was employed for estimating the predictive accuracies of individual and consensus models developed with the modelling dataset. Details about chemical structures, experimental values and references pertaining to the modelling and external validation sets are given in Table S1 of the Supplementary Materials.

### 2.2. Calculation of Descriptors

The calculation of the molecular descriptors of mixtures like DES requires special treatment so that these descriptors may account for structural/physicochemical attributes of each component as well as their molar ratios [23,24]. Previously, Oprisue et al. reported QSPR models for the density of a large number of mixtures [25]. In the same work, the authors described simple but effective calculation methodologies for binary mixtures. Among these, the ‘weighted by molar fraction mixture descriptors’ (henceforth referred as WM descriptors) must be noted; in our earlier studies, we found them highly useful to characterize DES properties [7,24]. In the present work, the WM descriptors may be classified into two types, namely *D*_pmix_ and *D*_nmix_, which were calculated according to Equations (1) and (2), below [25].

(1) $$D_{\mathrm{pmix}}=x_{1}D_{1}+x_{2}D_{2}$$

(2) $$D_{\mathrm{nmix}}=\;|x_{1}D_{1}-x_{2}D_{2}|$$

Following this strategy, descriptors of individual components (Descriptors *D*_1_ and *D*_2_ for HBA/cationic part of HBA and HBD, respectively) were weighted as per their molar fractions (*x*_1_ and *x*_2_ for components 1 and 2, respectively). The starting descriptors *D*_1_ and *D*_2_ are 2D descriptors, calculated with the Dragon software [26], which was accessed free of cost from the OCHEM webserver [25]. In fact, 3D descriptors were discarded, since reliable 3D conformations of DES components in the mixture demand high-level computational methods. Additionally, the widespread, exclusive use of *the most stable molecular conformation* yielded systematically erroneous descriptor values with misleading information for the inferred structure/property relationships [27]. Apart from these WM descriptors, three other independent variables were included: the measuring temperature, *T*(K), the presence/absence of chlorine ions, and the presence/absence of bromine ions. The latter two self-explanatory descriptors were binary (1/0) indicator variables that simply accounted for the composition of the DES’ HBA component. The inclusion of these two binary parameters was required; the WM descriptors were calculated only on the basis of the HBA’s cationic portion, with the contributions of the anionic part excluded. Calculations of WM descriptors from the starting descriptors were performed using our in-house software tool, QSAR-Mx, available under public license in https://github.com/ncordeirfcup/QSAR-Mx.

### 2.3. Dataset Division and Validation Methods

Similar to the descriptor calculation techniques, the dataset division demanded an advanced strategy. Indeed, any random division of datasets may give rise to underfitted and unreliable cheminformatic models [23,28]. Validation methods for mixtures that largely depend on the dataset division were described in detail by Muratov et al. [23,28]. Briefly, three unique dataset division and validation techniques—namely, points-out, (PO), mixtures-out (MO), and compounds-out (CO)—were introduced in the referred works. In PO, mixture data points are randomly distributed in such a way that each mixture is present in both the training and test sets. In the case of MO, mixtures are distributed in such a way that some mixtures are present in the training set and the rest of the mixtures are placed in the test set. Therefore, each mixture is present either in the training set or in the test set, but never in both sets. For CO, at least one compound of the dataset is never placed in the training set. Among these techniques, PO-based validation was found to be the weakest and should be avoided, whereas the CO technique was deemed the strongest validation strategy. Clearly, the utilization and goals of the mixtures-out- and compounds-out-based validation strategies are different [23]. The MO-based validation technique is the most suitable for predicting a mixture property. Therefore, this validation may be sufficient when the modelling dataset possesses a large structural heterogeneity. However, in practice, the model is expected to also be applicable to datasets containing new chemical entities. For example, the external validation set employed in the present work contained new compounds in either the HBA or HBD component of DES. The CO-based validation technique can ensure better predictivity in such cases, when the anticipated mixture is formed by a novel pure compound absent in the modelling dataset [24,28]. Thus, the CO-based validation is considered the most robust technique for mixtures. In this work, we attempted to set up models by applying both these validation strategies. At the same time, we employed a consensus prediction analysis with the highly predictive models resulting from both MO- and CO-based validations.

**Figure 1:**
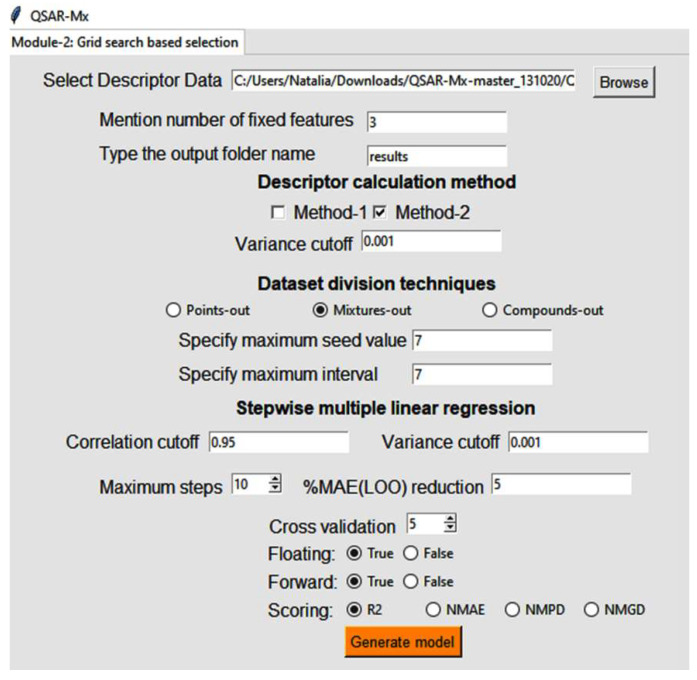
Screenshot of the in-house, publicly accessible tool QSAR-Mx, used for setting up the presented QSPR models.

Nonetheless, it should be noted here that neither MO- nor CO-based validation is straightforward; indeed, any unsystematic selection of the validation set based on these techniques may not yield the most predictive model. This is especially true in the case of linear QSPR modelling, for which feature selection is largely conditioned by the training data. Therefore, our in-house tool QSAR-Mx was designed to produce QSPR models with multiple automatically-generated MO- and CO-based data-distributions. In so doing, the most suitable data distribution and the most predictive model can be easily identified by means of statistical metrics. The functionalities of QSAR-Mx have been detailed in the instruction manual, which is accessible from https://github.com/ncordeirfcup/QSAR-Mx. Shortly, this tool requires two user-specific parameters—seed and interval—for setting up multiple data distributions based on the mixtures-out and compounds-out validation techniques. In the MO technique, the tool (i) identifies unique mixtures present in the dataset and (ii) sorts them, considering their number of instances in descending order. From the sorted list, the sample mixtures are collected according to the seed (the starting point for selection) and interval values. The selected unique mixtures are then placed in the test set. In Module 2 of QSAR-Mx (see screenshot of Figure 1), the user can input the maximum values for seed and interval chosen, and the data distributions are created by iterating all values between 1 and those values. Similarly, for the CO technique, the *QSAR-Mx* tool starts to sort the unique chemicals that belong to component-1, followed by sorting them according to the number of instances in descending order and finally, by choosing some chemicals based on the maximum values of seed and interval given. The process is then repeated for the unique chemicals, which belong to component2. The selected unique chemicals comprise the test set. Note that QSAR-Mx always places the sample with the maximum number of instances in the training set. After selecting the data distributions, QSAR-Mx generates multiple linear regression (MLR) models for each of these distributions. Only models with a test set size reaching at least 20% of the modelling dataset size were considered in this work. The main advantage of the QSAR-Mx tool is that it provides a straightforward and one-directional strategy for linear model development using MO/CO-based validation techniques.

### 2.4. Feature Selection and Model Development

The linear interpretable models were developed employing sequential forward selection-based multiple linear regression (SFS-MLR) analysis. The current SFS-MLR modelling was performed using the Sequential Feature Selector module of Mlxtend (http://rasbt.github.io/mlxtend/) [29], implemented in our in-house QSAR-Mx tool. Multiple SFS-MLR models were generated by varying the following parameters:

(i) Scoring method: four scoring methods related to statistical parameters such as the determination coefficient (*R*^2^), negative mean absolute error (NMAE) and the negative mean Poisson deviance (NMPD) were used for model selection.

(ii) Cross-validation (CV): the possibility of using 5-fold, 10-fold or no CV was allowed.

A correlation cutoff of 0.95 was set to remove highly intercorrelated descriptors. During model development, selection of the optimal number of descriptors was guided through a scheme entitled %MAE_LOO_ reduction, implemented in QSAR-Mx. Initially, all models were generated with a maximum of 10 descriptors (by setting maximum steps to 10, see Figure 1). At the same time, %MAE_LOO_ reduction was fixed at 5, ensuring the inclusion of one descriptor in the model if its addition reduced the value of leave-one-out (LOO) cross-validated mean absolute error (MAE_LOO_) by at least 5% with respect to the existing model. Otherwise, further addition of descriptors is terminated immediately. Therefore, the %MAE_LOO_-based selection guaranteed incorporation of the optimal number of descriptors in the present QSPR models—i.e., no descriptors were force fed into the models. Simultaneously, this strategy helped to compare the predictive efficiencies of multiple QSPR models generated with different data distributions as well as model development criteria from a neutral condition. Still, if the best model had 10 descriptors, the maximum step was increased to 15 while keeping the %MAE_LOO_ reduction option at 5 in order to check for the possibility of inclusion of a greater number of descriptors. If additional descriptors were found to be viable, these were considered, albeit only if their inclusion into the model improved its external predictivity.

### 2.5. Model Evaluation

The best models were selected, taking into consideration, first of all, the internal validation parameters MAE_LOO_ and *Q*^2^_LOO_ (LOO cross-validated determination coefficient *R*^2^) [30]. Then, two additional external validation parameters were considered: the mean absolute error for the test set (MAEtest) and the variance explained in external prediction (*Q*^2^_F1_) [30,31]. Along with these frequently used statistical parameters, another internal prediction parameter—the so-called leave-chemical-out cross-validated *R*^2^ (*Q*^2^_LCO_)—was also addressed. *Q*^2^_LCO_ is a new criterion, conceptually similar to leave-many-out cross validation *R*^2^ (or *Q*^2^_LMO_); however, the removal of samples is more strategic than in the former. This technique is applicable only to binary mixtures. For the calculation of *Q*^2^_LCO_, all mixtures formed by a new chemical (with observed property *Y_i_*) that belonged to component-1 of the training dataset (HBAs in our case) were removed one by one. After each removal, their predicted values (*Ŷ*_L(HBA)O_) were obtained with the model derived using the remaining training set samples. A similar procedure was applied to each chemical belonging to component-2 (HBDs in our case) to obtain *Ŷ*_L(HBD)O_. The final parameter *Q*^2^_LCO_ was then calculated according to the following equation:

(3) $$Q^{2}{}_{\mathrm{LCO}}=\frac{\left(1-\frac{\sum_{i}{\left(Y_{i}-{\hat{Y}}_{L(\mathrm{HBA})O}\right)}^{2}}{\sum_{i}{\left(Y_{i}-Y_{m}\right)}^{2}}\right)+\left(1-\frac{\sum_{i}{\left(Y_{i}-{\hat{Y}}_{L(\mathrm{HBD})O}\right)}^{2}}{\sum_{i}{\left(Y_{i}-Y_{m}\right)}^{2}}\right)}{2}$$

where *Y_m_* is the average observed property for the training set samples. It may be inferred that, although *Q*^2^_LCO_ uses the idea of the well-known leave-many-out cross-validation approach [30], it can be particularly useful for the internal validation of models developed with mixtures.

Similarly, one more statistical parameter, MAE_LCO_ (leave-compounds-out based mean absolute error), was calculated as follows:

(4) $${\mathrm{MAE}}_{\mathrm{LCO}}=\frac{\left(\frac{\sum_{i}\left|Y_{i}-{\hat{Y}}_{L(\mathrm{HBA})O}\right|}{N}\right)+\left(\frac{\sum_{i}\left|Y_{i}-{\hat{Y}}_{L(\mathrm{HBD})O}\right|}{N}\right)}{2}$$

where *N* stands for the total number of datapoints of the training set. A large difference between the values of *Q*^2^_LOO_ and *Q*^2^_LCO_ (or MAE_LOO_ and MAE_LCO_) indicated that the model fitting for at least one component of the mixtures was not satisfactory. Such a model should be avoided as it can not satisfy the compounds-out cross-validation internal predictivity criteria. In addition to the above-mentioned statistics, the statistical significance of the final models was also checked by additional internal predictivity statistics, such as the absolute-average-relative-deviation (AARD), and two scaled *r_m_*^2^ metrics (i.e., *r_m_*^2^_LOO_ and ∆*r_m_*^2^. Essentially, *r_m_*^2^ metrics are based on the correlation between the observed and predicted values, with and without intercept for the least squares regression lines [32]. Correspondingly, the AARD_test_, along with the scaled parameters *r_m_*^2^_test_ and ∆*r_m_*^2^_test_, were used for external validation. A more detailed description of these statistical parameters can be found elsewhere [14,30,31,32,33]. One should note here that criteria based on the lowest AARD are uncommon in QSPR modelling. However, these are useful for understanding the statistical significance of the models developed for thermodynamic properties. Thus, we included such parameters, as these allowed us to compare the statistical quality of the models proposed here with that of previously developed ones [14].

The statistical robustness of the final model was established through the *Y*-randomization method. This method proceeded as follows: first, several new models were generated with randomized responses (resorting to the same set of variables) and then, the metric *^c^R*^2^*_P_* was calculated [34] by the following equation:

(5) $${}^{c}{R^{2}}_{P}=R\cdot \sqrt{\left(R^{2}-R_{r}{}^{2}\right)}$$

where *R*^2^ and *R_r_*^2^ stand for the determination coefficients of the original non-randomized model and the randomized model, respectively. Therefore, high values of *^c^R*^2^*_P_* (at least greater than 0.5) indicated that the original model was not obtained by chance.

Additionally, the applicability domain (AD) of the developed models was determined. To do so, we built the so-called Williams plot, in which standardized residuals were plotted against leverage values. Doing so permitted us to identify response and structural outliers [35,36]. All plots shown in the present work were conceived with Matplotlib [37].

### 2.6. Consensus Prediction with Multiple Models

The most predictive QSPR models generated with multiple data division techniques (MO- and CO-based) and development criteria were subjected to consensus modelling. For this purpose, the Intelligent Consensus Predictor software was utilized. The four following techniques were used as described by Roy et al. [38]:

(a) Consensus model 0 or original consensus: simple arithmetic average of predicted response values from all input individual models;

(b) Consensus model 1: simple arithmetic average of predictions from qualified individual models;

(c) Consensus model 2: weighted average predictions from all qualified models. In this method, a weightage value is assigned to a qualified model with respect to a specific test set sample and the average is then calculated from the weighted models;

(d) Consensus model 3: best selection of predictions (compound-wise) from qualified individual models. In the latter, the model with the least cross-validated MAE of ten compounds similar to a particular test compound is selected for prediction.

The efficacy of consensus modelling was estimated with respect to the external validation set. Then, structurally similar samples were identified with a threshold value equal to mean Euclidean distance plus three times the standard deviation of Euclidean distance (i.e., mean + 3*SD).

## 3. Results and Discussion

**Figure 2:**
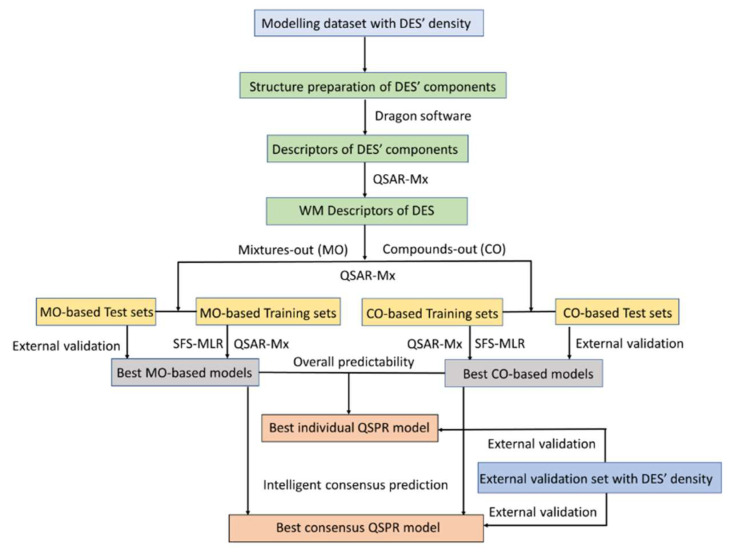
Basic workflow diagram for the QSPR analysis, adopted in this work.

Figure 2 shows a diagram illustrating the basic workflow followed in this work. Two of its major purposes were: (a) to identify the best individual model for characterization of the density of DES and (b) to identify the models for best consensus prediction. In order to obtain the best individual QSPR model, the most predictive models from both MO-based and CO-based data divisions were first determined separately and then compared.

Let us first consider the QSPR models generated with MO-based data divisions. A total of 90 models (MO1-MO90) were generated using QSAR-Mx, with maximum values of seed and interval set to 7. A summary of the statistical performance of all these models is given in Table S2. With different dataset division strategies and model development criteria, the statistical quality of such models varied to a considerable extent. After sorting the resulted models according to the lowest MAE_LOO_ values, 15 models with the most significant internal predictivities were identified. A summary of the statistical performance of these models is given in Table 1.

As may be expected, these fifteen MO-based models presented large variations in their external predictivity. Some of these models (for example, MO12, MO85, MO31 and MO71) were generated with high inter-collinearity among any of their two descriptors (R > 0.8). Overall, MO59 was selected as the best MO-based model, as it delivered the most significant statistical quality, judging from the high values of *Q*^2^_LOO_ (= 0.954) and *Q*^2^_LCO_ (= 0.919) and the low value of MAE_LOO_ (= 0.013). At the same time, this model, which was produced with 535 test set samples, gave rise to a satisfactory external predictivity, as follows from its metrics *R*^2^_Pred_ (= 0.748) and MAE_test_ (= 0.0328). Nevertheless, we checked whether the model could accept a higher number of descriptors by employing the 5% MAE_LOO_ reduction criterion. In so doing, we could have found a model with 11 descriptors by increasing the maximum step to 15, rather than using the initial value of 10. Yet, at the 11th step of stepwise selection, the reduction of MAE_LOO_ was less than 5%. In spite of having slightly higher internal predictivity (i.e., *Q*^2^_LOO_ = 0.957, *Q*^2^_LCO_ = 0.906 and MAE_LOO_ = 0.0128) *R*^2^_Pred_ and MAE_test_ of this eleven-descriptor model reduced to 0.741 and 0.0326, respectively. In other words, the additional descriptor failed to improve the external predictivity of the model. Therefore, the ten-descriptor model MO59 was retained as the final, and best, MO-based model.

Regarding the CO-based validation, the QSAR-Mx tool generated a total of 55 QSPR models (CO1-CO55, for details see Table S2). As in the previous case, the top 15 CO-based models were selected based on the lowest MAE_LOO_ values. A summary of the statistical performance of these models is shown in Table 2.

Similar to the derivation of MO-based models, the results, as presented in Table 2, clearly indicated that, with different data-distributions and model development strategies, the statistical quality of the MLR models varied significantly. Several models from Table 2, comparably to those from Table 1, showed a substantial level of inter-collinearity. Additionally, although some of the models presented rather high internal predictivity, their external predictivities were found to be unsatisfactory. Among all the CO-based models, model CO15 stood out due to its overall characteristics. The latter model was generated with 10 descriptors. Therefore, the %MAE(LOO) reduction rule was applied by increasing the maximum step to 15, as described for the case of MO-based models. However, this did not result in additional viable descriptors. Thus, the presented number of descriptors was considered optimal for model CO15. Moreover, the maximum inter-correlation between any of two descriptors was fairly small (*R* = 0.503), prompting independence among its descriptors. Thus, model CO15 appeared to be rather robust. The MO-based model MO59, however, exhibited a slightly higher, but still acceptable, inter-collinearity among descriptors (*R* = 0.776; see Table 1).

Equations and extended statistical results for both models CO15 and MO59 are provided in Table 3. As can be seen, the *Y*-randomization test performed with 1000 runs gave rise to *^c^R^2^_P_* values of 0.948 and 0.931 for models MO59 and CO15, respectively, suggesting that both of these were unique in nature. Noticeably, the MO59 model displayed better external predictivity as compared to the CO15 model (see MAE_test_ and %AARD_test_ values), although a greater number of test set samples were present in the former. As far as internal predictivity was concerned, both models yielded equivalent statistical results.

**Figure 3:**
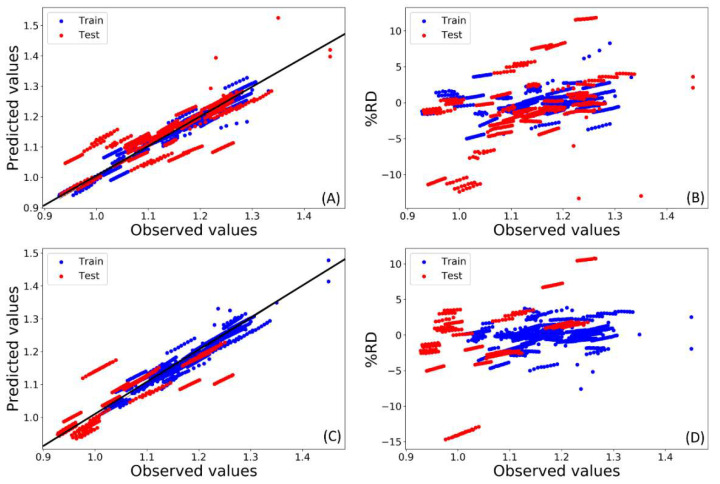
Plots of (**A**) predicted vs. observed density values for MO59, (**B**) percentage of relative deviation, %RD, vs. observed density values for MO59, (**C**) predicted vs. observed density values for CO15, (**D**) %RD vs. observed density values for CO15.

Figure 3 shows the plots of the predicted densities vs. the experimental observed densities, as well as the relative deviation percentage (%RD) vs. the experimental observed densities. As can be noted from this figure, the distribution of test set samples was somewhat clustered for CO15. Contrastingly, a more uniform distribution was obtained for MO59.

**Figure 4:**
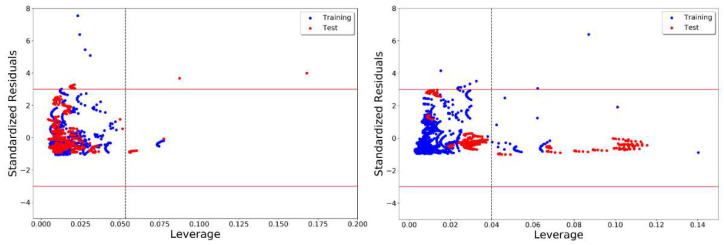
Williams plots of the best mixtures-out validation-based model, MO59 (**left**), and the best compounds-out validation-based model, CO15 (**right**).

To critically examine the predictivity of models MO59 and CO15, we compared their Williams plots [35,36], as presented in Figure 4. As expected, model CO15 had a larger number (129 with h* = 0.0399) of structural outliers as compared to model MO59 (25 with h* = 0.0533). On the other hand, the number of response outliers obtained (absolute SDR > 3) for models MO59 and CO15 were 19 and 10, respectively.

Figure 3 and Figure 4 present a typical scenario for MO- and CO-based validation approaches. In CO validation, new chemicals and their mixtures are placed in the test set to resort to a more rigorous validation strategy. Consequently, these test set samples might occupy a separate physicochemical space than the training set samples. For instance, in CO15, all mixtures containing tetrabutylammonium salts, L-proline, ethylene glycol, L-glutamic acid and propionic acid were placed in the test set. Unsurprisingly, more structural outliers were obtained in the corresponding Williams plot (Figure 4). However, most of these structural outliers were predicted remarkably well by CO15. This indicated a high efficiency of the model when predicting the density of DES prepared with new chemicals, which was the exact purpose of the compounds-out based validation.

Interestingly, MO59 placed as many mixtures as 17 chemicals (namely: citric acid, D-glucose, diethylamine, tetrahexylammonium salt, 1,2-propanediol, 2,3-butanediol, L-arginine, D-sucrose, L-glutamic acid, glycolic acid, mandelic acid, O-cresol, oxalic acid, p-chlorophenol, propionic acid, tartaric acid, and xylitol) exclusively in the test set. Therefore, model MO59 also satisfied the criteria for compounds-out validation. This arose from the MO-based data division procedure implemented in QSAR-Mx (see Materials and Methods), which ensured that only new mixtures assigned by the seed and interval values were placed in the test set. For large and diverse datasets, such a policy could produce some test mixtures composed by chemicals not present in the training set. In spite of including several new chemicals in the test set, MO59 yielded a smaller number of structural outliers. Thus, due to the significant structural diversity of both sets, model MO59 was considered the more reliable predictor.

Furthermore, 19 response outliers found in MO59 belonged to only five mixtures: trimethylglycine-2-chlorobenzoic acid (1:2), choline chloride-d-sucrose (1:1), choline chloride-d-sucrose (2:1), benzyl tripropylammonium chloride-oxalic acid (1:1), and tetrabutylammonium chloride-phenylacetic acid (1:2). The presence of the D-sucrose containing DES among the structural outliers may be explained by taking into account that D-sucrose was the only disaccharide present in the modelling dataset. Notwithstanding, removal of all sucrose-based DES from the modelling dataset only slightly improved the external predictivity of the model (MAE_test_ = 0.032, *R*^2^_Pred_ = 0.758, %AARD_test_ = 2.897). Therefore, these structural outliers were retained in the modelling dataset along with all other structural outliers predicted well by the model [39].

Hence, after considering all the aforementioned details as well as the better overall (internal plus external) predictivity, MO59 was selected as the best individual QSPR model. The descriptors of this model were used to understand crucial structural and physicochemical factors responsible for the density of DES. Yet the high predictivity of CO15 and other CO-based models should not be ignored. Consequently, highly predictive models obtained from both MO- and CO-based validation schemes were considered for consensus modelling, which will be discussed further. The performance characteristics of model MO59 against the modelling dataset (such as descriptor values, predicted density, outlier information, etc.) are shown in Table S3.

**Figure 5:**
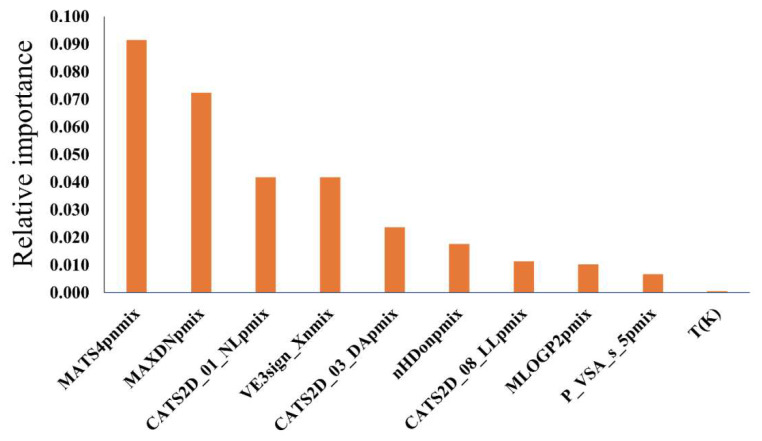
Relative importance of the descriptors found in the best individual model MO59.

Density is a physicochemical property and is generally difficult to interpret from molecular descriptors. The relative contributions of the descriptors of model MO59 are shown in Figure 5 with the help of a variable importance plot.

The absolute difference (*D*_nmix_ type) of weighted MATS4p descriptors between two components of a DES was found to have the highest importance in this QSPR model. MATS4p is a 2D autocorrelation descriptor conveying the Moran autocorrelation at a specific topological distance (lag-4), weighted by polarizability [40,41]. Importantly, the relationship between polarizability and density has now been well established [42]. As seen, MATS4p_nmix_ was positively correlated to density—meaning that the higher the values of this graph-based topological descriptor, the higher the DES density. What is more, since the Moran autocorrelation descriptors disclosed property deviations from average values, it can be inferred that the difference in polarizability between two DES components was related to the density of these components’ mixtures [43].

The sum (*D*_pmix_ type) of weighted MAXDN descriptors was found to be the second most influential descriptor. MAXDN, i.e., maximal electrotopological negative variation, is an E-state topological index encoding information regarding the effect on each atom due to the perturbation of its neighboring atoms [40,41]. This effect is based on the atomic intrinsic state (I), computed as the ratio between the Kier–Hall electronegativity of the atom and its number of bonds. MAXDN can be related to the nucleophilicity of the chemical species and, based its positive correlation with the density, it suggested that nucleophilic components would trigger denser DES.

The MO59 model contained three two-dimensional chemically advanced template search (CATS2D) descriptors [44]. Among them, CATS2D_01_NL_pmix_ exhibited the maximum relative importance in the model. CATS2D descriptors are topological descriptors that provide information regarding two types of atomic features at a given topological distance (lag) within the hydrogen-depleted molecular graph. As an example, CATS2D_01_NL accounted for both negative and lipophilic atomic features located at lag-1. Similarly, CATS2D_03_DA and CATS2D_08_LL represented hydrogen bond donor-acceptors at lag-3 and two lipophilic features at lag-8, respectively. CATS2D_08_LL_pmix_ showed negative correlation with density, contrarily to the other two CATS2D descriptors.

The fourth most important descriptor of the model was a 2D matrix-type descriptor entitled VE3sign_X, which stands for the logarithmic coefficient sum of the last eigenvector from the chi-matrix. Its positive correlation with the density indicated that the greater the absolute difference of the weighted descriptors between two DES’ components, the denser the DES will be.

Two descriptors, based on the number of hydrogen bond donors per mixture (nHDon) and the squared Moriguchi octanol-water partition coefficient (MLOGP2), were also found to impact the density of DES. Despite the low relative importance of MLOGP2pmix, it is one of the most frequently found descriptors in the SFS-QSPR models developed in this work. Clearly, this indicated that an increased number of hydrogen bond donor features and higher lipophilicity in the DES’ components could lead to a greater density of these solvents.

Another type of *D*_pmix_ descriptor, namely P_VSA_s_5, was found to contribute positively to DES density. P_VSA descriptors represent a comparatively novel type of descriptors that characterizes the amount of van der Waals surface area (VSA) having a property P in a certain range (at bin size 5 in this case) [45]. The property involved here was atomic intrinsic states, thus revealing once more the impact of both atomic electronegativities and their topological position within the DES’ components on DES density.

As a final descriptor, model MO59 included the influence of temperature (*T*(K)) on density. It is well known that, with increases in temperature, the density of these solvents gradually decreases. Similar to MLOGP2_pmix_, *T*(K) frequently appeared in the QSPR models developed here. While the latter descriptor contributed relatively little to the model, it clearly demonstrated the effect of temperature on DES density.

**Figure 6:**
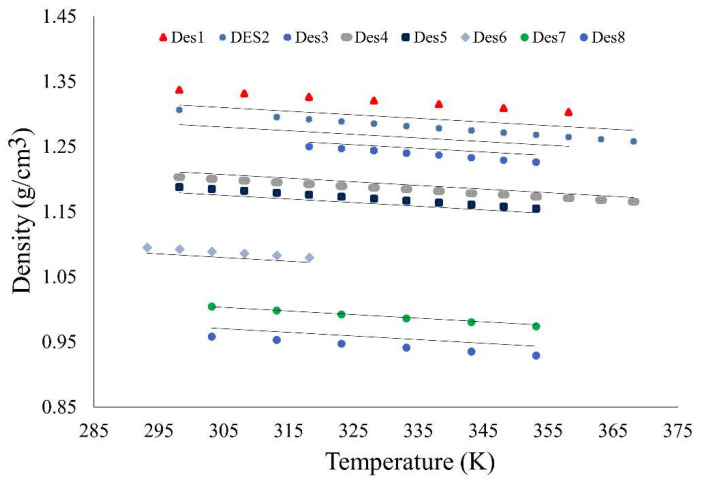
Comparison of densities calculated by the MO59 model to data in the literature, in temperature range from 283.15 K to 373.15 K for eight random DES at atmospheric pressure. DES1: choline chloride-d-fructose (1:1), DES2: methyltriphenyl phosphonium bromide-glycerol (1:2), DES3: acetylcholine chloride-D-fructose (1:1), DES4: choline chloride-glycerol (1:3), DES5: choline chloride-glutaric acid (1:1), DES6: choline chloride-phenol (1:3), DES7: tetrabutylammonium chloride-L-arginine (7:1), DES8: tetrabutylammonium chloride-L-aspartic acid (11:1).

The overall performance of model MO59 is illustrated in Figure 6, where the density values for eight randomly selected DES, taken from the literature and predicted by that model, were depicted in a wide range of temperatures. Th results proved that the proposed model was able to correlate temperature differences well with variation in DES density.

To sum up, our attempts to develop linear interpretable models gave rise to multiple QSPR models with comparable significant predictivities. Such highly predictive models could be used for consensus prediction as long as a separate dataset was available to estimate their predictive accuracies. Accordingly, the external validation set containing density data of 207 DES was employed for this purpose. It should be noted that none of the external dataset samples were included in the modelling dataset. Thus, such external datasets can be considered an ideal dataset for understanding the predictive efficiency of individual models as well as of intelligent consensus prediction. Initially, the three best models obtained from both from MO- and CO-based validation techniques (i.e., six in total) were selected for consensus prediction. The criteria for selection were the average values of MAE_LOO_ and MAE_test_, as well as reasonable levels of inter-collinearity (i.e., models with *R* > 0.80 between any two descriptors were discarded). In such a way, models MO75, MO59, MO10, CO15, CO17 and CO54 were chosen. Subsequently, the predictivity of these models was tested against the external validation set. The results for this external validation set are summarized in Table 4.

**Figure 7:**
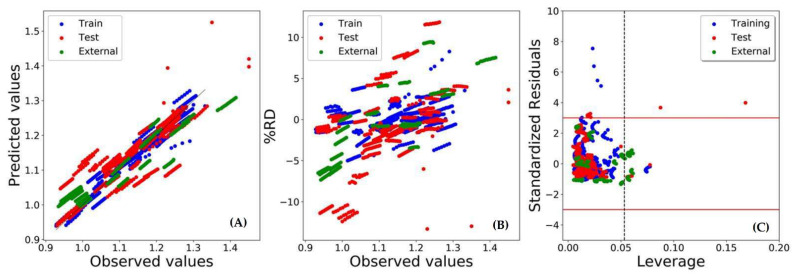
Plots for MO59 model against training, test and external validation sets: (**A**) observed vs. predicted values, (**B**) %RD vs. observed values, and (**C**) William’s plot.

All these QSPR models, save for MO10, presented high predictivity towards the external validation set. Regarding model MO10, its MAE_test_ value, being greater than 0.5, suggested a rather modest efficiency. Both models MO59 and CO15, which were identified in this work as the most predictive QSPR models, displayed similarly satisfactory predictivity against the external validation set. The external validation parameters of the best individual model (MO59) were: *R*^2^_Pred_ = 0.856, MAE_test_ = 0.041, *r_m_*^2^_(test)_ = 0.654, ∆*r_m_*^2^_(test)_ = 0.136, and %AARTD_test_ = 3.703. Figure 7 shows a comparison of the predicted *vs.* observed densities, as well as of the %RD vs. the observed densities for the best MO59 model and its final William plot. Significantly, 46 structural outliers (h* = 0.0533) were found in the external validation set, yet no detected response outlier (absolute SDR > 3) reiterated the high predictive efficiency of this model. After inspecting the outliers, we found that all these outliers contained 3-amino-1-propanol as HBD. Thus, the absence of this compound in the modelling dataset should be the main reason for their occurrence as structural outliers. Details on the MO59 prediction against the external validation dataset (i.e., descriptor values, predicted density and outlier information) are shown in Table S3.

Five models (namely, CO15, CO17, CO54, MO59 and MO75) showing MAE_test_ of less than 0.50 and AARD_test_ value of less than 4 against the external validation set were selected for consensus prediction. Evidently, these models consolidated good overall predictivity against both the external validation set and the modelling dataset. The equations and statistical parameters of CO17, CO54 and MO75 models are provided as Supplementary Material (Table S5). Interestingly, CO54 and MO75 comprised 7 and 5 descriptors, respectively. In other words, even with a comparatively small number of descriptors and, consequently, less internal predictivity, these two models revealed good predictivity against both the test and external validation sets. The overall predictivity of model CO17 was found to be similar to that of model CO15. In addition, 7 out of 10 descriptors of these two models were the same. It was noteworthy that, in addition to *T*(K), the lipophilicity-based descriptors, such as ALOGP_pmix_ and MLOGP2_pmix_, were consistently encountered in all these models, implying that the presence of hydrophobic constituents increased the density of DES.

The five best-performing models were combined into an intelligent consensus model in order to obtain the maximum predictive accuracy against the external validation set. The results of these experiments are shown in Table 5. First, all of the consensus models, C1–C11, helped to improve predictions toward the external validation set. Among all these models, model C9 had exceptionally excellent statistics with *R*^2^_Pred_ value of 0.921, MAE_test_ of 0.025 and %AARD_test_ of 2.151. This model was set up using three individual models, namely, CO54, MO75 and CO17, following a procedure where sample-wise predictions were made from qualified individual models [38]. All in all, model C9 was proposed for the prediction of the new DES’ density. Detailed results of this consensus prediction are provided in Table S5.

## 4. Conclusions

In this work, a systematic cheminformatics modelling analysis was carried out, with the aim of efficiently modelling the density of a large number of DES, following the principles of OECD guidelines. The individual models were set up with our in-house tool QSAR-Mx, which is a user-friendly, Python-based code that is available in public domain. Similarly, the consensus prediction models were derived with the help of an open access tool, Intelligent Consensus Predictor. Therefore, all proposed models are easily reproducible. Initially, the models were generated with a modelling dataset, previously used for development of simple and global thermodynamic model for estimating the density of DES [14]. It is important to mention that a number of thermodynamic models were reported to characterize the density of DES in the last decade [46,47,48,49,50]. Some recently published review articles also provided detailed descriptions about different thermodynamic modelling approaches for DESs [51,52]. Nevertheless, many of these models were developed with a small number of data points, as compared to our larger modelling dataset. Additionally, these models may not be considered proper QSPR models since they lacked a robust validation strategy, inspection of their applicability domain, and mechanistic interpretation from the context of molecular structures. The results of this work showed that cheminformatic methodologies may be considered an efficient alternative for delivering simple, global, and accurate models for estimating the density of DES. This work was further extended forward—predicting an external validation set collected from recently reported experimental density data. This external validation set allowed us to infer the predictive accuracies of the developed individual and consensus models. Though it was difficult to select the best individual QSPR model (since several of these displayed analogous predictive capacities), model MO59 was chosen on the basis of its high predictivity on the modelling dataset. The descriptors of this model were considered the most significant for characterizing the density of DES. The best individual model yielded an overall %AARD of 2.589, indicating that the performance of this QSPR model was better than that of the previously developed thermodynamic model (%AARD = 3.12) [14]. Upon analysis of this individual model, it was found that the lipophilicity, number of hydrogen bond donors per mixture, polarizability, van der Waals surface area, and topology of DES’ components all play important roles in determining the DES’ density.

This work provided valuable information regarding the structural attributes required for estimating the density of DES. It also laid out important guidelines for developing linear interpretable models with mixtures using rigorous validation techniques. Furthermore, the high predictivity obtained from consensus models toward the external validation set indicated that multiple models generated in the current study were highly effective at obtaining reliable predictions for novel DES.

## Figures and Tables

**Table 1:** Summary of statistical performance of the top 15 models (according to MAE_LOO_ values) obtained from MO-based data divisions.

Model	Model Development Parameters				Training Set Results				Test Set Results			Max Inc ^#^
	Scoring	CV	Seed	Intv ^*^	*N*_tr_	*Q* ^2^_LOO_	*Q* ^2^_LCO_	MAE_LOO_	*N*_test_	*R* ^2^_Pred_	MAE_test_	
MO029	NMAE	0	4	2	666	0.967	0.930	0.010	488	0.627	0.043	0.606
MO023	NMAE	0	2	2	619	0.967	0.930	0.010	535	0.626	0.040	0.586
MO011	*R* ^2^	0	4	2	666	0.973	0.949	0.0101	488	0.424	0.054	0.475
MO041	NMPD	0	2	2	619	0.972	0.955	0.010	535	0.527	0.046	0.573
MO035	NMAE	0	6	2	711	0.964	0.941	0.011	443	0.540	0.046	0.600
MO005	*R* ^2^	0	2	2	619	0.966	0.946	0.012	535	0.480	0.047	0.720
MO071	*R* ^2^	5	6	2	711	0.956	0.933	0.013	443	0.642	0.045	0.811
**MO059 †**	***R* ^2^**	**5**	**2**	**2**	**619**	**0.954**	**0.919**	**0.013**	**535**	**0.748**	**0.033**	**0.776**
MO012	*R* ^2^	0	4	3	818	0.956	0.917	0.013	336	0.750	0.036	0.814
MO053	NMPD	0	6	2	711	0.952	0.936	0.014	443	0.444	0.052	0.719
MO017	*R* ^2^	0	6	2	711	0.953	0.933	0.014	443	0.475	0.050	0.719
MO085	*R* ^2^	10	4	4	894	0.943	0.924	0.014	260	0.543	0.050	0.841
MO047	NMPD	0	4	2	666	0.951	0.929	0.014	488	0.503	0.046	0.715
MO031	NMAE	0	4	4	894	0.926	0.902	0.015	260	0.658	0.043	0.918
MO022	NMAE	0	1	5	915	0.940	0.919	0.016	239	0.689	0.033	0.643

^*^ Interval, ^#^ Maximum intercorrelation between any two descriptors. **†** Most predictive model is marked in bold.

**Table 2:** Summary of statistical performance of the top 15 models (according to MAE_LOO_ values) obtained from CO-based data divisions.

Model	Model Development Parameters				Training Set Results				Test Set Results			Max Inc ^#^
	Scoring	CV	Seed	Intv ^*^	*N*_tr_	*Q* ^2^_LOO_	*Q* ^2^_LCO_	MAE_LOO_	*N*_test_	*R* ^2^_Pred_	MAE_test_	
CO023	NMPD	0	1	1	609	0.947	0.901	0.010	545	0.637	0.063	0.647
CO001	*R* ^2^	0	1	1	609	0.948	0.875	0.011	545	0.624	0.054	0.541
CO012	NMAE	0	1	1	609	0.925	0.838	0.011	545	0.225	0.088	0.545
CO013	NMAE	0	1	2	825	0.956	0.934	0.012	329	0.750	0.055	0.535
CO014	NMAE	0	1	3	784	0.926	0.726	0.012	370	−3.107	0.168	0.931
**CO015 †**	**NMAE**	**0**	**1**	**4**	**827**	**0.934**	**0.915**	**0.012**	**327**	**0.867**	**0.036**	**0.503**
CO004	*R* ^2^	0	1	4	827	0.938	0.927	0.013	327	0.731	0.060	0.503
CO026	NMPD	0	1	4	827	0.938	0.927	0.013	327	0.731	0.060	0.503
CO016	NMAE	0	1	5	831	0.930	0.900	0.013	323	0.618	0.068	0.868
CO002	*R* ^2^	0	1	2	825	0.950	0.895	0.013	329	0.707	0.059	0.848
CO017	NMAE	0	1	6	837	0.927	0.891	0.014	317	0.880	0.041	0.538
CO005	*R* ^2^	0	1	5	831	0.931	0.910	0.014	323	0.625	0.068	0.833
CO027	NMPD	0	1	5	831	0.918	0.887	0.014	323	0.327	0.084	0.670
CO029	NMPD	0	2	1	600	0.954	0.925	0.014	554	0.645	0.045	0.852
CO018	NMAE	0	2	1	600	0.938	0.877	0.015	554	0.617	0.050	0.384

^*^ Interval, ^#^ Maximum inter-correlation between any two descriptors, **†** Most predictive model is marked in bold.

**Table 3:** Best models derived for the DES’ density (*ρ* in g/cm^3^) along with their MLR statistical parameters, using MO- and CO-based techniques (models MO59 and CO15).

Model	Equation	Training Set Results	Test Set Results
		*N*_training_ = 619; *R*^2^ = 0.956;	
MO59	*ρ* = +1.065(±0.012) + 0.072(±0.002) MAXDN_pmix_ + 0.007(±0.000) P_VSA_s_5_pmix_	*R*^2^_Adj_ = 0.955;	*N*_test_ = 535;
	+0.018(±0.002) nHDon_pmix_ + 0.024(±0.002) CATS2D_03_DA_pmix_	*F*(10,608) = 1305.70;	*R*^2^_Pred_ = 0.748;
	+0.042(±0.003) CATS2D_01_NL_pmix_ − 0.011(±0.006) CATS2D_08_LL_pmix_	*Q*^2^_LOO_ = 0.953; MAE_LOO_ = 0.013;	MAE_test_ = 0.033,
	+0.010(±0.000) MLOGP2_pmix_ + 0.042(±0.002) VE3sign_X_nmix_	*Q*^2^_LCO_ = 0.919; MAE_LCO_ = 0.018;	*r_m_*^2^_(test)_ = 0.646;
	+0.091(±0.009) MATS4_pmix_ − 0.001(±0.000) *T*(K)	*r_m_*^2^_(LOO)_ = 0.933; ∆*r_m_*^2^_(LOO)_ = 0.040;	∆*r_m_*^2^_(test)_ = 0.199;
		%AARD_training_ = 1.151;	%AARD_test_ = 2.914
		*^c^R*^2^*_P_* (1000 runs)= 0.948	
		*N*_training_ = 827; *R*^2^ = 0.937;	
CO15	*ρ* = +1.101(±0.014) + 0.033(±0.002) AMW_pmix_ − 0.066(±0.005) Psi_i_1d_pmix_	*R*^2^_Adj_ = 0.936;	*N*_test_ = 327;
	−0.012(±0.000) ATSC8m_pmix_ + 0.851(±0.016) ATSC1e_pmix_	*F*(10,816) = 1213;	*R*^2^_Pred_ = 0.867;
	−0.255(±0.016) VE2_Dz(Z)_pmix_ + 0.054(±0.005) nCconj_pmix_	*Q*^2^_LOO_ = 0.934; MAE_LOO_ = 0.012;	MAE_test_ = 0.036;
	−0.029(±0.002) CATS2D_02_DL_pmix_ + 0.010(±0.000) MLOGP2_pmix_	*Q*^2^_LCO_ = 0.915; MAE_LCO_ = 0.014;	*r_m_*^2^_(test)_ = 0.586;
	+0.185 (±0.014) GGI5_nmix_ − 0.001(±0.000) *T*(K)	*r_m_*^2^_(LOO)_ = 0.905; ∆*r_m_*^2^_(LOO)_ = 0.055;	∆*r_m_*^2^_(test)_ = 0.205;
		%AARD_training_ = 1.040;	%AARD_test_ = 3.400
		*^c^R*^2^*_P_* (1000 runs) = 0.931	

**Table 4:** Summary of the performance of the best three MO-based and best three CO-based QSPR models (sorted by the MAE_test_ values) obtained for the external validation set.

Model		Parameters			Training Set				Test Set			External Validation Set		
	Scoring	CV	Seed	Intv	*N*_tr_	*Q* ^2^_LOO_	*Q* ^2^_LCO_	MAE_LOO_	*N*_ts_	*R* ^2^_Pred_	MAE_test_	*N*_ex_	*R* ^2^_Pred_	MAE_test_
CO54	*R* ^2^	10	4	1	854	0.881	0.838	0.025	300	0.803	0.030	207	0.867	0.034
MO75	*R* ^2^	10	1	4	856	0.865	0.845	0.025	298	0.802	0.020	207	0.879	0.038
CO17	NMAE	0	1	6	837	0.927	0.891	0.014	317	0.880	0.041	207	0.874	0.039
CO15	NMAE	0	1	4	827	0.934	0.915	0.012	327	0.867	0.036	207	0.842	0.040
MO59	*R* ^2^	5	2	2	619	0.954	0.919	0.013	535	0.748	0.033	207	0.856	0.041
MO10	*R* ^2^	0	3	4	885	0.884	0.865	0.022	269	0.903	0.022	207	0.786	0.051

**Table 5:** Results obtained for the external validation set (*n* = 207) by consensus prediction using the most significant QSPR models. The best consensus model is marked in bold.

No.	Models					CM	*R* ^2^_Pred_	MAE_test_	*r* *_m_* ^2^_(test)_	∆*r_m_*^2^_(test)_	%AARD_test_
C1	CO54	MO75	CO17	CO15	MO59	2	0.903	0.030	0.883	0.046	2.544
C2	CO54	-	CO17	CO15	MO59	2	0.901	0.300	0.895	0.047	2.533
C3	CO54	MO75	CO17	CO15	-	3	0.906	0.027	0.918	0.038	2.281
C4	CO54	MO75	-	CO15	MO59	2	0.898	0.031	0.840	0.057	2.592
C5	CO054	MO75	CO17	-	MO59	2	0.911	0.029	0.868	0.050	2.460
C6	-	MO75	CO17	CO15	MO59	0	0.893	0.033	0.850	0.057	2.813
C7	CO54	-	CO17	CO15	-	3	0.906	0.027	0.916	0.036	2.301
C8	CO54	MO75	-	CO15	-	3	0.893	0.028	0.903	0.017	2.311
**C9**	**CO54**	**MO75**	**CO17**	**-**	**-**	**3**	**0.921**	**0.025**	**0.932**	**0.031**	**2.151**
C10	CO54	-	CO017	-	-	3	0.921	0.026	0.929	0.029	2.171
C11	CO54	MO75	-	-	-	3	0.907	0.030	0.793	0.074	2.619

## Data Availability

Further details about the data presented in this study are available on request from the corresponding authors.

## Supplementary Materials

The following are available online: Table S1. List of DES and experimental density data; Table S2. Summary of the statistical performance of all CO- and MO-based models; Table S3. Summary of the results for the best model found; Table S4. Detailed results of the consensus prediction; Table S5. Models CO54, MO75 and CO17, derived for DES’ density (*ρ* in g/cm^3^), along with their statistical parameters.

## Sample Availability

Not available.
