# Supplementary material for: Density of Deep Eutectic Solvents: The Path Forward Cheminformatics-Driven Reliable Predictions for Mixtures
Source: Molecules. 2021 Sep 24;26(19):5779. doi: 10.3390/molecules26195779 (PMC8510218; doi:10.3390/molecules26195779)
Supplement: Supplementary file 1 [file molecules-26-05779-s001.zip › TableS5.pdf]

Density of deep eutectic solvents: The path forward cheminformatics-driven reliable predictions for mixtures

Amit Kumar Halder[a], Reza Haghbakhsh[b,c], Iuliia V. Voroshvlova[a], Ana Rita C. Duarte[c], M. Natalia D. S. Cordeiro[a*]

[a]LAQV@REQUIMTE/Department of Chemistry and Biochemistry, Faculty of Sciences, University of Porto, 4169-007 Porto, Portugal
[b]School of Chemical and Petroleum Engineering, Shiraz University, Mollasadra Ave., Shiraz, 71348-51154, Iran
[c]LAQV@REQUIMTE/Departament of Chemistry, Faculty of Sciences and Technology, New University of Lisbon, 2829-516, Caparica, Portugal

* Correspondence: ncordeir@fc.up.pt

Table S5. Models CO54, MO75 and CO17 derived for the DES' density (ρ in g/cm^3) along with their statistical parameters.

Model	Equation	Training set results	Test set results
CO54	$\rho = +1.180(\pm 0.020) - 0.083(\pm 0.003)\text{TI2\_L}_{pmix} + 0.348(\pm 0.022)\text{SM1\_Dz(Z)}_{pmix} + 0.027(\pm 0.001)\text{Eta\_F}_{pmix} - 0.028(\pm 0.001)\text{ALOGP}_{pmix} - 0.112(\pm 0.006)\text{rGes}_{nmix} + 0.059(\pm 0.006)\text{SpMin2\_Bh(s)}_{pmix} - 0.0004(\pm 0.0000)T(\text{K})$	$N_{training}$ = 854; R^2 = 0.883; R^2_{Adj} = 0.882; $F(7,846)$ = 914.3; Q^2_{LOO} = 0.881; MAE_{LOO} = 0.025; $r_m^2{}_{(LOO)}$ = 0.831; $\Delta r_m^2{}_{(LOO)}$ = 0.099; Q^2_{LCO} =0.838; MAE_{LCO} = 0.030; $\%AARD_{tr}$ = 2.186	N_{test} = 300; R^2_{Pred} = 0.803; MAE_{test} =0.030, $r_m^2{}_{(test)}$ = 0.662; $\Delta r_m^2{}_{(test)}$ = 0.114; $\%AARD_{test}$ = 2.491
MO75	$\rho = +1.120(\pm 0.021) - 0.020(\pm 0.003)\text{S3K}_{pmix} + 0.346(\pm 0.012)\text{SM1\_Dz(m)}_{pmix} + 0.028(\pm 0.002)\text{SpMax\_EA(dm)}_{pmix} + 0.013(\pm 0.000)\text{MLOGP2}_{pmix} - 0.0004(\pm 0.0000)T(\text{K})$	$N_{training}$ = 856; R^2 = 0.867; R^2_{Adj} = 0.866; $F(5,850)$ = 914.3; Q^2_{LOO} =0.865; MAE_{LOO} = 0.025; $r_m^2{}_{(LOO)}$ = 0.807; $\Delta r_m^2{}_{(LOO)}$ = 0.113; Q^2_{LCO} =0.844; MAE_{LCO} = 0.027; $\%AARD_{tr}$ = 2.246	N_{test} = 298; R^2_{Pred} = 0.802; MAE_{test} =0.028, $r_m^2{}_{(test)}$ = 0.726; $\Delta r_m^2{}_{(test)}$ = 0.101; $\%AARD_{test}$ = 1.750
CO17	$\rho = +0.913(\pm 0.013) + 0.044(\pm 0.001)\text{AMW}_{pmix} - 0.055(\pm 0.006)\text{Psi\_i\_1d}_{pmix} + 0.032(\pm 0.002)\text{SpMax7\_Bh(e)}_{pmix} + 0.119(\pm 0.010)\text{nRNHR}_{pmix} + 0.010(\pm 0.000)\text{MLOGP2}_{pmix} + 0.013(\pm 0.001)\text{SM13\_}A\text{EA(dm)}_{nmix} - 0.011(\pm 0.000)\text{ATSC8m}_{pmix} + 0.741(\pm 0.019)\text{ATSC1e}_{pmix} - 0.032(\pm 0.002)\text{CATS2D\_02\_DL}_{nmix} - 0.001(\pm 0.0000)T(\text{K})$	$N_{training}$ = 837; R^2 = 0.930; R^2_{Adj} = 0.929; $F(10,826)$ = 1095; Q^2_{LOO} = 0.927; MAE_{LOO} = 0.014; $r_m^2{}_{(LOO)}$ = 0.895; $\Delta r_m^2{}_{(LOO)}$ = 0.062; Q^2_{LCO} = 0.891; MAE_{LCO} = 0.017; $\%AARD_{train}$ = 1.136	N_{test} = 317; R^2_{Pred} = 0.879; MAE_{test} =0.041; $r_m^2{}_{(test)}$ = 0.627; $\Delta r_m^2{}_{(test)}$ = 0.116; $\%AARD_{test}$ = 3.927
